# Polysaccharides as the Sensing Material for Metal Ion Detection-Based Optical Sensor Applications

**DOI:** 10.3390/s20143924

**Published:** 2020-07-15

**Authors:** Nur Hidayah Azeman, Norhana Arsad, Ahmad Ashrif A Bakar

**Affiliations:** Photonics Technology Laboratory, Department of Electrical, Electronic and Systems Engineering, Faculty of Engineering and Built Environment, Universiti Kebangsaan Malaysia, UKM Bangi 43600, Selangor, Malaysia; noa@ukm.edu.my

**Keywords:** polysaccharides, chitosan, carrageenan, sensing interaction, optical sensors

## Abstract

The incorporation of a proper sensing material towards the construction of high selectivity optical sensing devices is vital. Polysaccharides, such as chitosan and carrageenan, are among the bio-based sensing materials that are extensively employed due to their remarkable physicochemical attributes. This paper highlights the critical aspects of the design of suitable polysaccharides for the recognition of specific analytes through physical and chemical modifications of polysaccharide structure. Such modifications lead to the enhancement of physicochemical properties of polysaccharides and optical sensor performance. Chitosan and carrageenan are two materials that possess excellent features which are capable of sensing target analytes via various interactions. The interaction between polysaccharides and analytes is dependent on the availability of functional groups in their structure. The integration of polysaccharides with various optical sensing techniques further improves optical sensor performance. The application of polysaccharides as sensing materials in various optical sensing techniques is also highlighted, particularly for metal ion sensing.

## 1. Introduction

The application of sensing materials in the construction of sensor devices is critical to enhancing sensor performance. Numerous kinds of sensing materials, such as polymer [1], nanomaterials [2] and composites [3,4] have been synthesized for specific sensor applications. The physicochemical properties of sensing materials can be further improved by modifying the structure of sensing materials, either by physical or chemical methods [5]. Researchers opted to explore the natural bio-based sensing material for various sensor applications, due to the increasing cost of synthetic resins and material waste disposal issues [6,7].

Polysaccharide is a biopolymeric sensing material that has excellent physicochemical properties. It is formed by repeating units and joined by glycoside linkages with various degrees of branching [8]. The general formula of polysaccharides is (C_6_H_10_O_5_)*_n_*, where *n* is in the range of 40 to 3000 [9]. Interestingly, different sources of polysaccharides produce various characteristics, such as chemical compositions, molecular weights, chemical structures, surface properties and extensive networks of intra- and inter-hydrogen bonding [9,10]. It is essential to understand the origins and features of polysaccharides before designing a selective sensing materials-based polysaccharide for specific target analytes. Table 1 lists the generic polysaccharides according to their origins.

Chitosan and carrageenan are among the two most abundant natural polysaccharides that exist on Earth. Chitosan is derived from crustacean shells, while carrageenan is derived from red seaweeds (marine red algae) of the Rhodophyceae class. The main difference between these two polysaccharides are the functional groups in their structure. Chitosan possesses O-H, N-H and C-O functional groups, whereas carrageenan possesses O=S=O, O-H and C-O functional groups. These two materials have good potential in optical sensor applications with their own unique sensing performance [11]. An extensive review on the usage of chitosan for photonics and nanomaterials research, which highlighted optical material properties and mechanisms, is well documented in [12].

This article reviews the important features in designing chitosan and carrageenan as the sensing material for optical sensor technology. The general background, physicochemical properties, physical and chemical interaction of the target analyte with the sensing materials are highlighted. A report on several recent optical sensing technologies employing polysaccharides as the sensing material is also summarized. A comprehensive review on the physical and chemical modification of polysaccharides had been highlighted in the work published in [13,14], thus it is not covered in this review.

### 1.1. Chitosan

Chitosan, which originates from the exoskeletons of shellfish and crustaceans, was first discovered by Rouget in the year 1859 through the deacetylation of chitin [15]. It is known as a linear polysaccharide which composed of β-(1–4)-2-acetamido-D-glucose and β-(1–4)-2-amino-D-glucose units [16]. It is non-toxic, biodegradable and has good anti-microbial characteristics [17,18]. Chitosan is insoluble in solvents but can be dissolved in weak acidic media at pH < 6.5 and exists as a positively charged compound at this pH value [12]. At this condition, chitosan is prone to interact with another negatively charged compounds, leading to electrostatic interaction. Furthermore, the solubility of chitosan is dependent on the degree of deacetylation, such as the distribution of acetyl groups along the main chain, its molecular weight and the properties of the acid used for protonation [19]. Other than that, the molecular weight of chitosan also affects its quality in terms of elasticity and brittleness [20,21].

The adsorption features of chitosan is influenced by several factors: (i) the large number of hydroxyl groups in chitosan affects its hydrophilicity, (ii) the number of primary amino groups in chitosan leads to high active adsorption sites and (iii) the flexible structure of chitosan’s polymer chain allows it to adopt a suitable configuration for complexation with ions [22]. 

**Figure 1:**
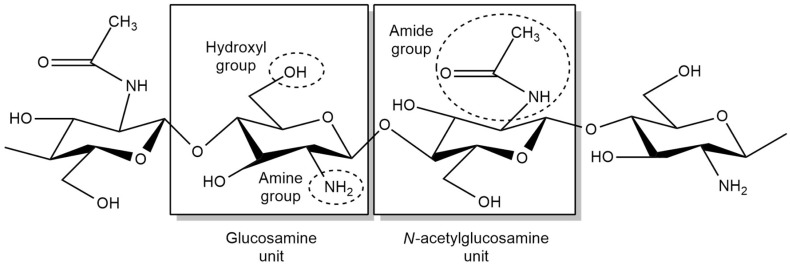
Chemical structure of chitosan.

As shown in Figure 1, chitosan possesses amino and hydroxyl functional groups. These functional groups are responsible for cation binding due to the presence of lone pairs of electrons on nitrogen and oxygen atoms. However, amino groups have a higher tendency to donate electrons and chelate with metal cations due to a weaker attraction of a lone pair towards its nucleus in comparison to an oxygen atom. Besides, a higher adsorption ability of chitosan is due to the three-dimensional chelating structure of chitosan in comparison to a planar surface [23]. Hence, chitosan has been used in wastewater treatment, ion exchangers and functional matrixes [24]. On top of that, chitosan can be easily transformed into a film via the solution casting method, whereby the film possesses high mechanical strength, good thermal stability, excellent homogeneity and a smooth surface, which makes it a suitable material for sensor applications [25]. 

### 1.2. Carrageenan

Carrageenan is derived from a red seaweed of the class Rhodophyceae. It is known as a sulfated polysaccharide which consists of 15% to 40% ester sulfate content and has an average relative molecular mass above 100 kDa. Carrageenan is formed by alternate units of D-galactose and 3,6-anhydro-galactose (3,6-AG) joined by α-1,3 and β-1,4-glycosidic linkages. Carrageenan is classified based on the composition and degree of sulfation at specific locations in its structure, namely lambda (λ), kappa (κ) and iota (ι) carrageenan. The sulfate groups are covalently bonded to the carbon atoms C-2, C-4 or C-6 of individual galactose residues via ester linkages. The sulfate content varies for different types of carrageenan, resulting in highly negatively charged polymers. Higher levels of ester sulfate mean lower solubility and lower gel strength [26,27,28].

Kappa-carrageenan has an ester sulfate content of about 25% to 30% and a 3,6-anhydrogalactose content of about 28% to 35%. Meanwhile, iota-carrageenan has an ester sulfate content of about 28% to 30% and a 3,6-anhydrogalactose content of about 25% to 30%. Lambda-carrageenan has an ester sulfate content of about 32 to 39% and no content of 3,6-anhydrogalactose [29]. Theoretically, a higher content of sulfate in carrageenan increases the number of electronegative atoms that are able to interact with the target analyte, resulting in sensitivity enhancement. Carrageenan has been used in various applications, such as in food preparation, pharmaceutical applications [30] and as anti-inflammatory agents [31]. 

**Figure 2:**
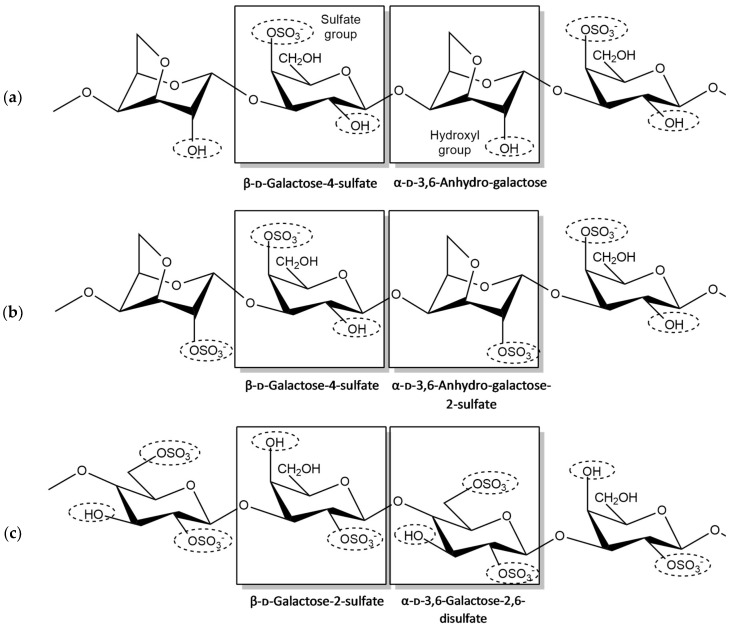
Chemical structure of (**a**) kappa, (**b**) iota and (**c**) lambda-carrageenan.

Figure 2 illustrates the basic functional groups that are present in the structure of carrageenan: hydroxyl and sulfate, where sulfate possesses an additional oxygen atom in its structure, which is good for cation chelation [1]. As seen in Figure 2, the number of sulfates in each carrageenan is different. While kappa-carrageenan has one sulfate group, iota- and lambda-carrageenan have two and three sulfate groups, respectively. 

## 2. Interaction between Polysaccharides and Chemical Analytes

To sense the chemical analytes, polysaccharides have to be designed in such a way that allows them to interact with the target chemical analytes. The interaction between polysaccharides and chemical analytes relies upon the content of functional groups in the polysaccharide structure. Some of the possible interactions, for example, are chelation, electrostatic interaction and hydrogen bonding. It is important to note that deacetylation degree, crystallinity and the molecular weight of polysaccharides influences the interaction and sorption properties of polysaccharides with analytes as well [32].

### 2.1. Chelation

Chelation is the ability of a compound to interact or form coordinate bonding with other ions or ligands. This phenomenon is dependent on the availability of empty molecular orbitals and usually occurs at a nearly neutral pH [32]. Such interaction is greatly influenced by the pH of the solution due to the competition of protons with other ions. The interaction mechanism between the sensing material and analyte can be studied using high-end instruments such as UV-spectrophotometry, infrared spectrometry and potentiometry [32,33]. In general, the chelation mechanism of polysaccharides with analytes is innumerable depending on the functional groups that are present in the polysaccharides.

**Figure 3:**
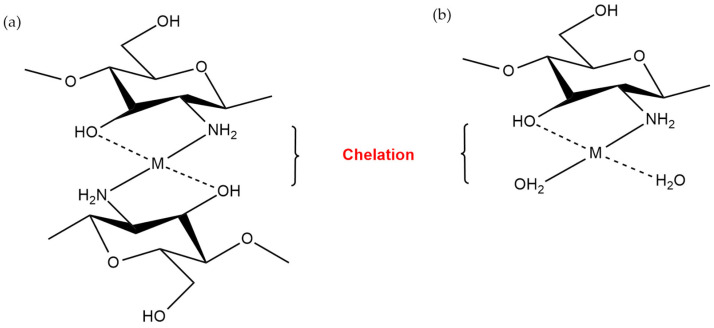
Possible metal chelation interaction between chitosan and an analyte in (**a**) bridge and (**b**) pendant models [34].

For instance, in chitosan, amine groups represent the most active functional groups that are responsible for metal ion chelation due to the presence of an electron lone pair on the nitrogen atom [33]. The chelation mechanism in chitosan can be classified into two types: (i) the bridge and (ii) the pendant models. When the metal ions are bound with several amine groups via inter- or intramolecular complexation, it is known as the bridge model (Figure 3a). On the contrary, the pendant model is classified when the amine groups of chitosan form a chelation with a metal ion in a pendant manner (Figure 3b) [32]. Based on the hard and soft acid and base theory, amine groups are classified as “intermediary” ligands, where they possess greater affinity towards transition metal ions and soft-acid metals in comparison to alkaline, alkaline earth metals and hard-acid metals [33]. 

On the other hand, for carrageenan, sulfates are accountable for ion chelation [35]. A recent study by Cao and co-workers [36] shows that the chelation of carrageenan with a metal ion is affected by several factors, including valency and the hydration size of the metal ion. For example, κ-carrageenan has one negative sulfate group, which allows it to selectively bind with a monovalent metal ion, such as K^+^, Cs^+^ or Rb^+^. Meanwhile, ι-carrageenan and λ-carrageenan, with two and three negatively charged sulfate groups, respectively, have a tendency to selectively bind with a divalent metal ion, Ca^2+^, and a trivalent metal ion, Fe^3+^, respectively. 

### 2.2. Electrostatic Interaction

The electrostatic interaction occurs when the sensing material and analyte are attracted to one another due to the charge difference of the ion or compound. For instance, in an acidic solution, the amine group of chitosan is protonated, producing a positively charged cationic compound, and hence opens possibilities to bind with a negatively charged anionic compound. In this condition, chitosan is able to attract an anionic compound by electrostatic interaction. In contrast with that of the chelation mechanism in the previous section, the metal binding properties via electrostatic interaction are not influenced by the environment of the amine groups. Because, in this case, the charge of the protonated amine groups is responsible for metal anion uptake [33]. Correspondingly, the sorption capability of chitosan is also strongly dependent on the pH of the solution, however, the effect of pH primarily influences the competition between the target and interference analytes for sorption. 

**Figure 4:**
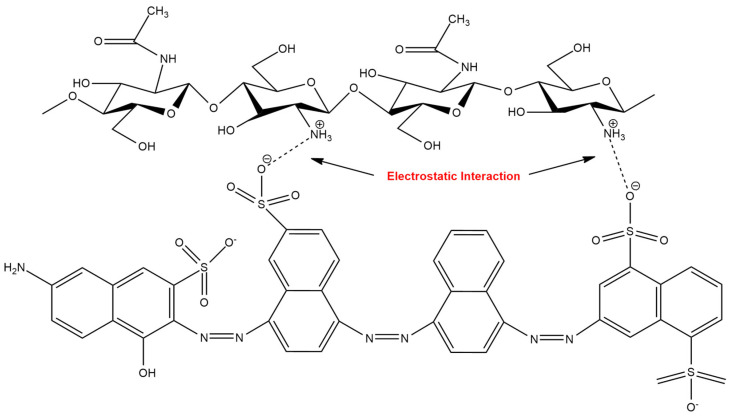
Possible electrostatic interaction between chitosan and dye analyte [38].

Abdullah and co-workers [1] developed a localized surface plasmon resonance (LSPR) technique based on chitosan and carrageenan as sensing materials for the detection of Pb^2+^. According to their findings, carrageenan is able to form more electrostatic interactions with Pb^2+^ than chitosan, resulting in high sensitivity. This is due to the presence of additional electronegative atoms in carrageenan, which contributes its functional group. In another report, Mobarak and co-workers [37] synthesized carboxymethyl carrageenan and studied the chemical interaction properties of carboxymethyl carrageenan with Li^+^. According to their discoveries, the electrostatic interaction between Li^+^ and the carboxylic groups of carboxymethyl carrageenan is more favorable than for the sulfate group. This could be explained by a higher basic property of the carboxylic group compared to the sulfate group. The general example of electrostatic interaction between chitosan and an analyte is depicted in Figure 4.

### 2.3. Hydrogen Bonding

**Figure 5:**
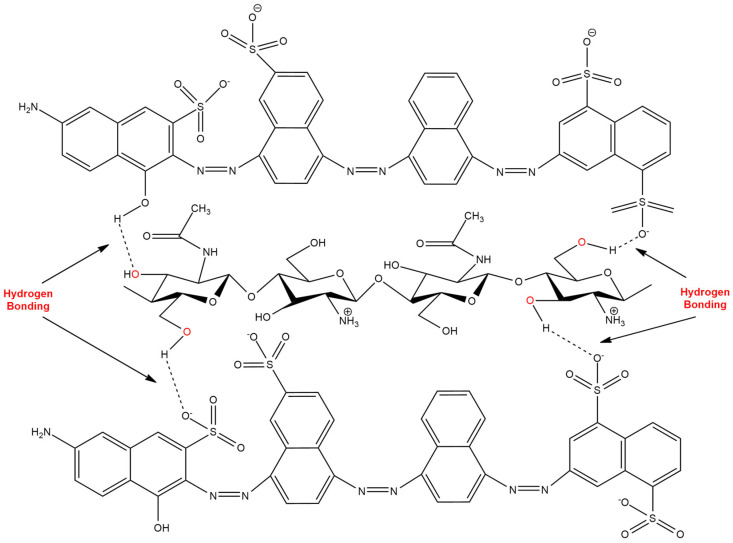
Possible hydrogen bonding between chitosan and dye analyte [38].

When two molecules have a different polarity, they are attracted with one another via electrostatic interactions. Hydrogen bonds can be described as the electrostatic interactions that occur specifically between hydrogen with highly electronegative atoms, such as fluorine, oxygen and nitrogen [39]. For instance, Pirillo and co-workers [40] investigated the use of chitosan as an adsorbent for dye removal in aqueous solutions. Their findings suggest that the factor affecting the adsorption properties of chitosan is the nature of the target analyte itself. An analyte surface that is rich in hydroxyl groups is able to interact with the oxygen atom of chitosan. Figure 5 shows the hydrogen bonding interaction between chitosan and a commercial dye [39]. 

It is worth highlighting that the interactions between polysaccharides and analytes through metal ion chelation, electrostatic interaction and hydrogen bonding are dependent on the fraction of deacetylated units, polymer chain length, crystallinity, molecular weight, polymer conditions, the physical form of polysaccharides, pH and the composition of the solution, as well as the selectivity and binding affinity of polysaccharides towards target analytes [22].

## 3. Deposition of Polysaccharide Thin Film for Optical Sensor Applications

The selection of a deposition technique for sensing material during sensor device fabrication is crucial to ensure the attachment and distribution of sensing material on the substrate surface, thus allowing the sensor to perform efficiently. There are ample ways to deposit sensing material on the sensor device surface, such as sol–gel dip-coating, spin-coating and layer-by-layer assembly techniques.

### 3.1. Dip-Coating Technique

**Figure 6:**
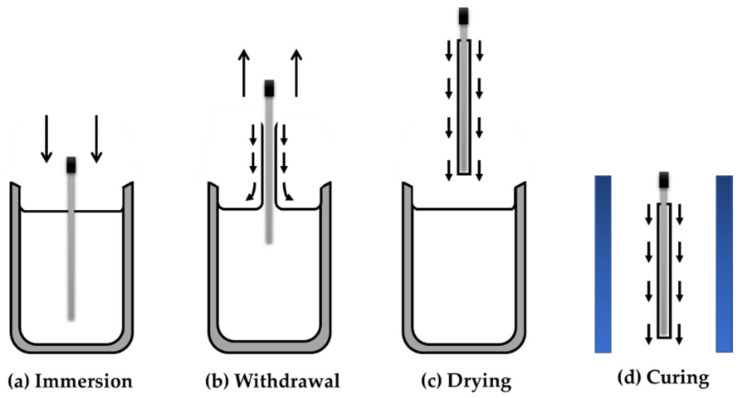
Material deposition using sol–gel dip-coating technique, (**a**) immersion, (**b**) withdrawal, (**c**) drying and (**d**) curing of substrate.

The dip-coating technique is the most common and simple deposition technique for coating sensing material on the sensor device. This technique was first pioneered in the year 1940 by Schott and was widely applied during the 1950s in industry [41]. The dip-coating technique can be described as a wet liquid film deposition by the withdrawal of a substrate from a liquid coating medium [41]. There are four stages that are carried out when using this technique. Firstly, the substrate of either glass substrate or optical fiber is immersed into a sensing material solution. Here, the substrate is dipped for a while, allowing the attachment of sensing material on the substrate surface. Secondly, the substrate is pulled out from the solution to remove excess sensing material on the surface of the substrate, allowing the formation of a thin film. Finally, the formed thin film is dried under a certain temperature according to the thermal properties of the sensing material. Under certain conditions, a post-treatment step is necessary for the final production of the thin film. Figure 6 shows the summary of the dip-coating process for the production of thin films [41]. 

Ni and co-workers [42] deposited chitosan on the surface of optical fiber using a dip-coating technique. In their work, chitosan was dissolved in 4% acetic acid, which causes the protonation of many amino groups in its structure, leading to the formation of positive charges in the chitosan structure. Therefore, to coat positively charged chitosan on an optical fiber surface, the optical fiber was first treated with piranha solution for at least 45 min to achieve a negatively charged surface. Then, the optical fiber was dipped into the chitosan solution at a rate of 30 mm/min by a stepper motor. The process was repeated thrice to achieve a smooth and uniform coating on the optical fiber surface. Subsequently, the optical fiber was dried at 45 °C for 2 h. The thickness of the coated material produced using this method was not reported. 

In another report, Yulianti and co-workers [43] conducted a coating process by dipping the fiber tip into a chitosan solution for 10 s, followed by drying process at room temperature for 2 h. Chitosan was prepared by dissolving it in 1% of acetic acid and was stirred using a magnetic stirrer for 3.5 h at 90 °C. The process was repeated 15 times to obtain layers of the desired thickness. The coat thickness produced was analyzed using an optical microscope and was reported to be 141.85 μm, 105.52 μm and 129.25 μm for three different sensors, respectively. It can be summarized that the dip-coating rate, replication process and drying parameters affect the attachment and thickness of the polysaccharides coated on the optical fiber surface. Researchers determined these parameters based on their study applications. Another point to be highlighted is that, although the dip-coating process parameters are fixed, in most cases, it is still difficult to obtain a uniform thickness or consistent thickness value of coating due to gravity-assisted flow during the process. 

### 3.2. Spin-Coating Technique

**Figure 7:**
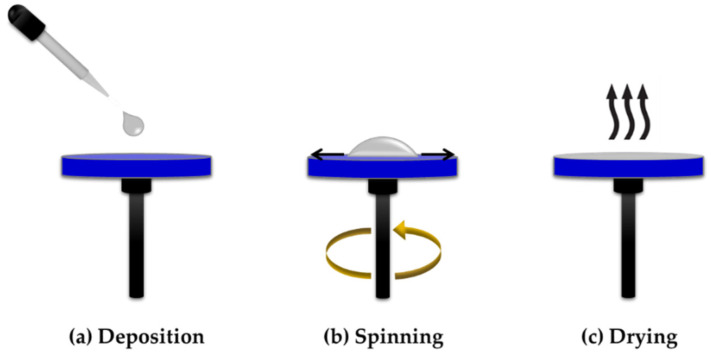
Material deposition using spin-coating technique, (**a**) deposition, (**b**) spinning and (**c**) drying of substrate.

Spin-coating is commonly used for depositing sensing material on a flat surface substrate [44]. Material deposition is carried out by dropping material on the substrate surface and rotating the substrate using a spin-coater machine. The physics behind the substrate rotation is the condition of fluid flow where the rotational accelerations are exactly balanced by the viscous drag felt within the solution. In the spin-coating technique, there are two major factors that affect the distribution of the sensing material on the substrate, which are viscous flow rate and the evaporation rate. The viscous flow rate is normally controlled by the rotation speed, while on the other hand, the evaporation rate is controlled by the heating or drying condition. By controlling these two factors, a uniform and well-distributed sensing material could be obtained. Typically, the coating thickness produced by the spin-coating technique is below 1 micron [44]. However, the drawback of using this technique is that it wastes a lot of material during the spinning process. There are plenty of works published on the use of the spin-coating technique for sensing material deposition in LSPR [1] and surface plasmon resonance (SPR) [3,7] applications. Figure 7 shows the step-by-step material deposition method using a spin-coating technique.

### 3.3. Layer-By-Layer Assembly (LBL)

Another deposition technique is layer-by-layer (LBL) assembly, which is also commonly known as the electrostatic self-assembly technique. It is reported that the LBL technique is an effective method for functionalization on fiber surfaces [45]. Raghunandhan and co-workers [45] deposited chitosan/polyacrylic acid on an optical fiber surface via the LBL assembly technique for the detection of metal ions. The fiber was first treated with acids prior to the deposition of chitosan using a dip-coating technique. Then, the fiber was dried and washed with deionized water to remove residues on the optical fiber surface. The same procedure was repeated for polyacrylic acid. This constitutes a complete bilayer deposition cycle. The functionalized fiber was then allowed to dry overnight in a vacuum oven at 60 °C. It is worth noting that treating the substrate or optical fiber with acids prior to sensing material deposition is not necessary for glass substrates or optical fibers that are coated with metal. This is to avoid the removal of thin films from the surface of the substrate [46]. 

**Figure 8:**
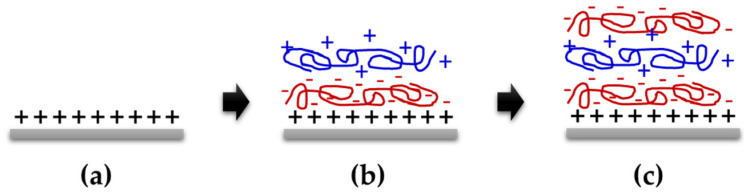
Material deposition using layer-by-layer assembly technique, (**a**) treatment with acid, (**b**) chitosan and polyacrylic acid depositions (completed bilayer deposition cycle) and (**c**) repeating the same deposition procedure alternately.

In other report, Mironenko and co-workers [47] deposited chitosan/carrageenan on a flat substrate using an LBL technique for the optical detection of ammonia (NH_3_) and hydrochloric acid (HCl) gases. The substrate was prepared by cleaning with an H_2_O_2_/NH_3_/H_2_O (1:1:1, *v*/*v*/*v*) mixture at 80 °C in an ultrasonic bath, then thoroughly washing with distilled water prior to drying at 120 °C. Subsequently, the substrate was immersed in chitosan and carrageenan solutions alternately for 10 min each with an intermediate careful rinsing under running deionized water. Topography analysis shows that this method is suitable for optical waveguide sensing layers, as it produced a homogeneous surface coverage and slightly thinner coatings, which can prevent scattering losses of optical waveguides. Figure 8 shows a step-by-step process for material deposition using the layer-by-layer assembly technique. 

## 4. Application of Polysaccharides as Sensing Material in Optical Sensor Technology

### 4.1. Colorimetric Optical Sensing Using Polysaccharide-Based Materials

Colorimetric optical sensing is one of the simplest techniques in optical sensor applications owing to its simplicity, low cost, miniaturization of size and lack of any additional instruments. A colorimetric sensor can be described as a visual detection instrument that shows color change upon the interaction of a sensing material with a target analyte [48]. To construct a colorimetric sensor, the involvement of a material with excellent optical properties, such as organic dyes and nanomaterials, is important to develop a distinct color change that can be observed visually. Quantum dots (QDs) are among the nanomaterials that have been applied in colorimetric sensing due to their outstanding advantages over traditional dyes, such as bright photoluminescence (PL), continuous excited profiles, a narrow emission range and high photostability [49]. Ma and co-workers [49] fabricated novel carboxymethyl chitosan-coated CdTe QDs (CMCS/CdTe-QDs) for the detection of Zn^2+^ in prostate cancer cells. The sensing mechanism is based on the compatibility and strong binding ability of CMCS in a CMCS-CdTe-QD composite with Zn^2+^. Upon such a bond formation, a photoluminescence activation effect was induced and enhanced the PL of the QDs, which could be observed physically. By using this sensing material, Zn^2+^ can be detected in concentrations as low as 4.5 μM. In another report, Song et al. [50] developed CMCS-modified CdTe QDs and Zn^2+^ (CMCS/CdTe-QDs/Zn^2+^) for a colorimetric nanosensor for lysozyme detection. The sensing mechanism is based on the interaction of CMCS/CdTe-QDs/Zn^2+^ with lysozyme, where this interaction quenched the QDs’ PL. The developed method was highly selective and fast with a detection limit of 0.031 ng/mL. 

Bai and co-workers [51] improved the colorimetric sensing properties of carboxymethyl chitosan (CMCS) towards Al^3+^ by adding quercetin into its structure. The physicochemical properties of the developed new sensing material was evaluated prior to sensor performance analysis. A significant color change was observed due to the coordination bond formation between the hydroxyl group of quercetin with Al^3+^. A maximum sensitivity of 0.187 Abs^−1^ mg mL^−1^ was obtained when using a sensing material with the highest amount of quercetin mixed with CMCS. This is probably due to more active sites provided by the hydroxyl group of quercetin for Al^3+^ binding on its surface, leading to better sensitivity, compared to other CMCS–quercetin mixtures. 

Other types of nanomaterials that have been widely applied in colorimetric sensing and visual detection are gold (Au) and silver (Ag) nanoparticles, due to their superior optical properties of surface plasmon resonance (SPR). The plasmon resonance scattering of Au and Ag nanoparticles have been used for bio-affinity sensing. Basically, the sensing mechanism is based on molecular interactions on the surface of the substrate, which is functionalized with nanoparticles [48]. Amanulla and co-workers [52] synthesized a chitosan-stabilized gold nanoparticle-reduced graphene oxide (Chitosan/AuNP/GO) nanocomposite as a sensing material for the colorimetric detection of nitrite. An excellent selectivity was observed between the sensing material and nitrite ions due to the high specificity between the amines of chitosan in the chitosan/AuNP/GO nanocomposite and nitrous acid. The observed color change from wine red to purple indicated the interaction between the sensing material and nitrite. Nitrite was detected in concentrations as low as 0.1 μM in this work. Later, in 2019, the same research group in [53] published a colorimetric sensing technique for the detection of Hg^2+^ using chitosan-functionalized AuNPs assembled on sulfur-doped graphitic carbon nitride (chitosan/AuNP@S-g-C_3_N_4_). Excellent sensitivity and selectivity were obtained, with a detection limit of 0.275 nM.

Narayanan and Han [54] synthesized silver/silver chloride nanoparticles and incorporated them with carrageenan (Carr/Ag/AgCl) without using any toxic chemicals to produce a composite for Hg^2+^ ion sensing. The formation of crystalline AgNPs was observed after the AgNPs were bound with the hydroxyl and sulfate groups of carrageenan. The SPR sensing strategy is based on the specific binding of AgNPs in Carr-Ag/AgCl composites with Hg^2+^, which resulted in the reduction of Hg^2+^ to Hg^0^. This phenomenon caused the color of the solution to change from dark brown to white. It is worth noting that Carr-Ag/AgCl was unable to change the solution color of other heavy metal ions upon interaction, proving the high selectivity of this composite towards Hg^2+^. The sensor response shows a decline in SPR intensity, as the Hg^2+^ concentration increases, producing sensitivity and detection limits of 0.00318 abs μM^−1^ and 1 μM, respectively [54]. 

Meanwhile, in the work reported by Lobregas et al. [55], carrageenan-stabilized AgNPs were used for the colorimetric detection of Hg^2+^. The sensing mechanism is similar to the work reported in [54] via the interaction of AgNPs with Hg^2+^. The main difference in this work is the nature of the sensing material itself, where carrageenan is prepared in a hydrogel form. The limit of detection reported was 2.92 × 10^−4^ M. Recently, Wang and co-workers [56] prepared carrageenan-AgNPs for the dual colorimetric detection of Cu^2+^ and S^2−^. In their work, AgNPs were synthesized in a greener way using carrageenan as a reducing and capping agent. The interaction between carrageenan-AgNPs with Cu^2+^ and S^2-^ changed the color of solution from yellow to colorless and yellow to brown, respectively. The detection limit recorded for Cu^2+^ was 1.7 μM and 2 μM for S^2−^. Table 2 shows the summary of colorimetric optical sensing performance using polysaccharides. 

### 4.2. Surface Plasmon Resonance (SPR) Using Polysaccharide-Based Materials

Surface plasmon resonance (SPR) sensors were first discovered by Wood in 1902 [57]. He observed a pattern of “anomalous” dark and light bands in the reflected light when he shone polarized light on a mirror with a diffraction grating on its surface. Later, in 1968, Otto, Kretschmann and Raether reported on the surface plasmon excitation [57,58,59]. In 1983, Leidberg was the first scientist to discover molecular interaction using SPR sensors [60]. SPR can be defined as an optical sensor technique in which p-polarized light excites the charge density wave along the metal–dielectric interface by sustaining a resonance condition. SPR is highly sensitive, reliable and low-cost compared to other conventional optical sensor techniques [3]. 

**Figure 9:**
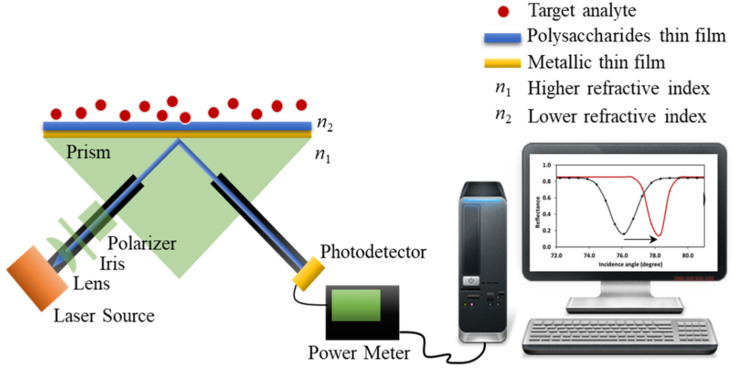
SPR experimental setup based on the Kretschmann configuration [3].

The traditional analysis of an SPR sensor is normally based on the full width at half maximum (FWHM) of SPR dips and shifts in SPR angles for different analytes. Both the SPR dip and the SPR shift are greatly influenced by the type of sensing material and the choice of metal that supports it. The majority of SPR applications use Kretschmann configuration, where a thin metallic film, such as Au or Ag, is placed at the interface of two dielectric media and are used for the excitation of surface plasmon waves on the SPR surface [3], as shown in Figure 9. As seen in Figure 9, medium 1 possesses a higher refractive index (*n*_1_) and medium 2 possesses a lower refractive index (*n*_2_). When p-polarized light travels from *n*_1_ to *n*_2_, the total internal reflection occurs in the medium when the incidence angle is greater than that of the critical angle leading to the formation of evanescent waves on the *n*_2_ surface [61]. The thickness of the metallic thin film that is used is critical, the SPR performance is optimum at a 50 nm thickness [7].

Since the SPR signal is based on the change of the refractive index of the medium, selectivity towards the target analyte is one of the major concerns for this technique. This limitation can be improved by modifying the SPR surface through the functionalization of ligands or coating with sensitive sensing materials [62,63]. Recently, the application of polysaccharides as sensing materials in SPR has been explored to improve the selectivity of the sensor. 

Mcllwee and co-workers, in 2008 [62], were some of the first to report on the use of polysaccharides as potential sensing materials for the detection of metal ions using the SPR technique. They used CS thin films for the SPR detection of Fe^3+^ ions. The optimum thickness of CS thin films for Fe^3+^ ion detection is in the range of 10 to 64 nm, with the lowest detection limit at 250 ppb. Later, in 2011, Fen and co-workers [63] reported on the synthesis and optical properties of crosslinked CS thin films with glutaraldehyde as crosslinkers for potential application in metal ion detection. Due to the chelating ability of CS towards positive charged metal ions, the performance of crosslinked chitosan for metal ion detection using the SPR technique was further studied. It is reported that the SPR sensitivity of Hg^2+^ and Cu^2+^ detection is 0.007 and 0.006 ppm^−1^, respectively, with the lowest detection limit at 500 ppb. 

**Figure 10:**
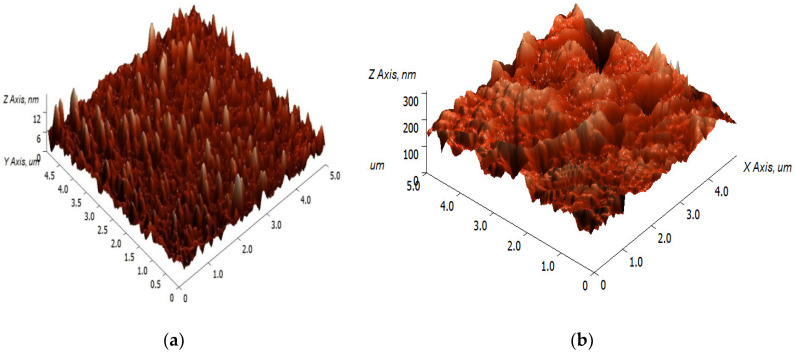
Atomic force microscopy (AFM) images of (**a**) before the addition of graphene oxide (GO) to chitosan (CS), surface roughness: 1.647 nm; and (**b**) after the addition of GO to CS, surface roughness: 31.040 nm [3].

To further improve the selectivity of CS towards target analytes, the immobilization of other chemicals, such as p-tert-butylcalix [4] arene-tetrakis [64] and tetrabutyl thiuram disulfide [65] in CS thin films for SPR sensors, was explored. Meanwhile, in 2014, Lokman et al. [66] developed an SPR technique based on an Au single layer coated with sensing material that comprised chitosan/GO (CS/GO) for the detection of Pb^2+^. Their findings suggest that the roughness of the surface of CS/GO analyzed by atomic force microscopy (AFM) increases after the addition of GO and exhibits the presence of additional functional groups, such as epoxy, hydroxyl and carboxyl, contributed by GO in the composite, as shown in Figure 10. The presence of GO in CS significantly improves mechanical interlocking with polymer chains, resulting a better adhesion of analytes on the thin film surface. In addition to that, the addition of GO to CS reduces the crystallinity properties of CS, indicating that CS and GO interact physically without modifying their chemical structures [66]. Because of the unique large surface area of GO, which allows the binding of more analyte, the SPR-based CS/GO nanocomposite shows better sensor performance, at about 1.112 ppm^−1^, in comparison to SPR-based CS with a sensitivity of 0.776 ppm^−1^. 

The selection of metallic layers during SPR fabrication is also crucial to obtaining a good SPR performance. It is reported that the application of Au in the SPR setup is more promising than Ag, due to its excellent stability and good sensitivity [7]. Although Ag gets oxidized easily, SPR-based Ag produces better detection accuracy compared to Au due to the larger permittivity negative value of Ag, hence producing narrower FWHM SPR spectra. Besides, the SPR sensor performance can also be improved by manipulating the structure of the metallic thin layer film used. Both Au and Ag metallic thin films have their own advantages and disadvantages in SPR applications. To overcome the drawbacks of these two thin films, researchers developed a multi-metallic structure of Au and Ag, whereas Au is normally coated on top of Ag to prevent the oxidation of the Ag thin film when exposed to air [7]. In the work reported by Kamaruddin and co-workers in 2014 [67] and 2016 [7], despite using a common SPR setup, which involved a single layer of Au metallic thin film, they developed an SPR technique based on Ag/Au bimetallic and Ag/Au/Ag trimetallic structured thin films for the detection of Pb^2+^ using a CS/GO nanocomposite as an active sensing layer. It was reported that the SPR performance based on the multi-metallic structure offers better evanescent field enhancement, great stability and remarkable sensing performance [7]. Later, in 2019, Lokman and co-workers [3] integrated CS/GO with Ag thin films, which further raised the SPR sensor performance in comparison to the work reported in [7], with 1.380° ppm^−1^. It can be deduced that incorporating GO in CS increases the surface roughness of the thin film, leading to a high surface-to-volume ratio, which is crucial for the attachment of analytes, leading to sensor performance enhancement [3,7]. 

Recently, hydroxyl- and carboxyl-functionalized graphene QDs were incorporated with chitosan derivatives for SPR optical sensor applications. Anas and co-workers [68] prepared a chitosan composite by incorporating hydroxyl-functionalized graphene QDs (CS/OH-GQDs) for the detection of Fe^3+^. The addition of chitosan to OH-GQDs increased the photoluminescence (PL) intensity of the composite material. Fe^3+^ has a high affinity towards CS/OH-GQDs with a binding affinity constant (K) of 5.79 ppm^−1^. This result is probably due to the high electronegativity of Fe^3+^, resulting in strong attraction towards the functional groups in CS/OH-GQDs. The positively charged of Fe^3+^ interacted with the negatively charged amino group in CS through the formation of shared electrons. It is reported that the SPR sensitivity and detection limit of 0.11396° ppm^−1^ and 0.5 ppm were obtained, respectively. In another report, Ramdzan and co-workers [69] incorporated carboxyl-functionalized graphene QDs with chitosan (CS/COOH-GQDs) and analyzed Hg^2+^ solutions with concentrations ranging from 0 to 100 ppm. The significant finding in this work is that the CS/COOH-GQDs coated on gold thin films increased the sensitivity of the SPR sensor. The sensing interaction occurred between the positively charged Hg^2+^ with the negatively charged amino group of chitosan in the CS/COOH-GQDs via electron sharing. The sensor sensitivity obtained was 0.00062° ppm^−1^. 

In summary, the SPR sensor performance is influenced by many factors, such as the selection of metallic thin films and the choice of sensing materials, as well as the configuration setup of SPR. By manipulating these factors wisely, an excellent sensor performance could be obtained. Apart from functionalizing the sensing material with specific functional groups for sensitivity and selectivity enhancement, the selectivity of the sensing material towards the target analyte is also dependent on the binding affinity of the analyte towards the sensing material. The binding affinity constant, K, that can be determined via isotherm study, provides quantitative information of binding interactions between analytes and sensing materials, which influence the selectivity of the sensor. On the other hand, pH and temperature are other parameters that affect the binding interaction between sensing materials and analytes as well [70]. Table 3 shows the summary of SPR performance based on polysaccharides. 

### 4.3. Localized Surface Plasmon Resonance (LSPR) Using Polysaccharide-Based Materials

Localized surface plasmon resonance (LSPR) is an optical technique that was pioneered by Michael Faraday in 1857 [71]. LSPR can be described as the collective electron oscillations at the interface of metallic nanoparticles that are excited by incident light of a specific wavelength [1]. Unlike SPR, metal nanoparticles are used in LSPR instead of metallic thin films. Typically, noble metals such as gold (AuNPs) and silver nanoparticles (AgNPs) are used in LSPR because of the d-d transition energy levels that exhibit LSPR in the visible range [72]. Although AgNPs exhibit stronger and sharper spectrum bands than AuNPs, AuNPs are favorable in most LSPR sensing techniques due to their inert nature [72]. Apart from improving the selectivity issue, researchers applied sensitive material, such as polymer, in LSPR for the stability improvement as well [73]. 

Many researchers selected CS due to its polycationic nature, which allows the attachment of metal nanoparticles on its surface via electrostatic interactions [73]. The application of CS for metal ion sensing using the LSPR technique was reported previously [1,73]. Praig and co-workers [74] prepared a crosslinked CS film by adding hexamethylene 1,6-di(aminocarboxysulfonate) as a crosslinker agent into a CS solution prior to integration with AuNPs. The as-prepared sensing material was used for the Fe^3+^ and Cu^2+^ metal ion detection using LSPR. The limits of detection reported for Fe^3+^ and Cu^2+^ ions were 0.5 µM and 0.5 mM, respectively [74].

**Figure 11:**
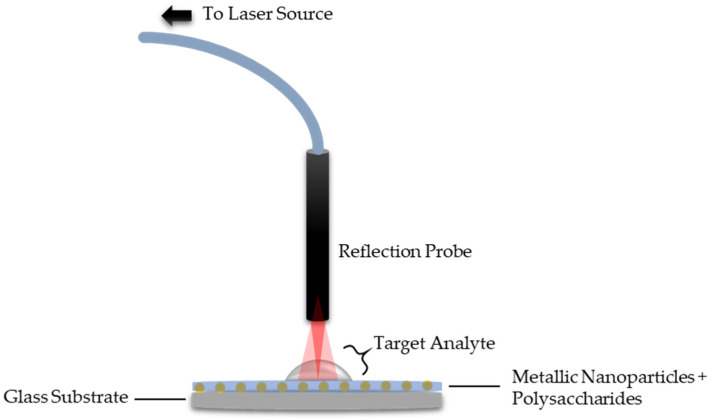
Localized surface plasmon resonance (LSPR) experimental setup.

In another report, Fahnestock and co-workers [75] developed an LSPR technique based on a CS/AuNP composite for the detection of Cr^6+^. The Cr^6+^ sensing was optimum in the pH ranging from 6.8 to 7.8, with the sensitivity and detection limits of 0.022 nm ppm^−1^ and 10 ppm, respectively [75]. In 2018, Abdullah et al. [1] reported on the application of CS and κ-carrageenan as the active layer for the detection of Pb^2+^ using a LSPR technique. LSPR-based κ-carrageenan exhibits nearly twice the enhancement in sensor performance compared to CS, with a sensitivity of 1.3535 nm^−1^ and 0.8165 nm^−1^, respectively. The increment in LSPR sensitivity is due to the presence of an additional electronegative atom in the κ-carrageenan structure, which provides additional adsorption sites for metal ion attachment on its surface [1]. Figure 11 and Table 4 show the schematic figure of the LSPR experimental setup and a summary of the LSPR technique based on polysaccharides, respectively.

### 4.4. Optical Fiber Sensors Using Polysaccharide-Based Materials

The vast advantages of using an optical fiber sensor technique for chemical and biosensing have been attested for the analysis of different analytes [2,76,77]. This technique is cost effective, resistant to electromagnetic interference and more importantly, it can be applied in situ. The structure of an optical fiber can be manipulated in numerous ways to produce different optical fiber sensing techniques, where each optical sensing technique produces a different sensor performance.

#### 4.4.1. Microfiber Sensor Using Polysaccharide-Based Materials

**Figure 12:**
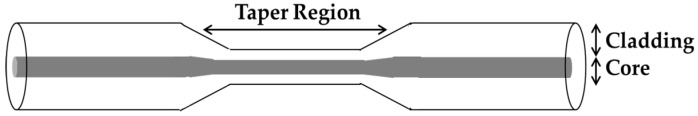
Structure of microfiber.

In chemical sensing and biosensing, the use of an ordinary optical fiber limits the interaction of propagating light with the target analyte due to the thick core-cladding diameter of the optical fiber, hence lowering the sensor sensitivity [2,76,77]. This limitation can be improved by tapering the optical fiber structure, hence producing a thinner core-cladding diameter of the optical fiber and enabling a high interaction between the propagating light and the chemical analyte, leading to higher sensor sensitivity. The integration of the sensing material at the tapered region of the optical fiber further improved the sensitivity of the sensors. Figure 12 shows the structure of the microfiber, which also known as a tapered optical fiber. 

The first report on the application of CS with a tapered optical fiber for Pb^2+^ ion sensing was published by Ibrahim and co-workers in 2016 [11]. The diameter of the core-cladding was reduced from 125 µm/62.5 µm to 20 µm and the length of the tapered region was 1 cm. The tapered region was functionalized with acid and base solutions prior to coating with CS. This procedure was carried out to improve the deposition of CS on the optical fiber surface. The sensor sensitivity obtained was 40.55 abs ppm^−1^ for Pb^2+^ concentrations ranging from 0.2 to 1 ppm. 

**Figure 13:**
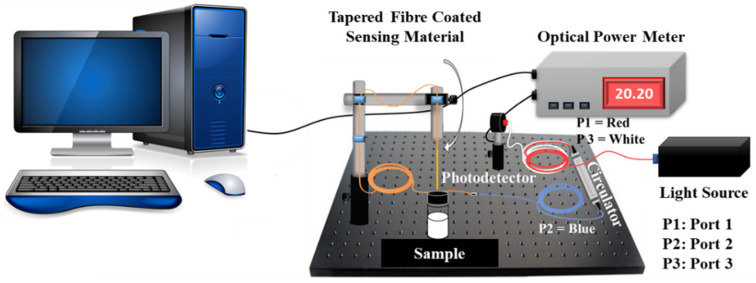
Experimental setup for optical fiber sensor–reflectance mode.

**Figure 14:**
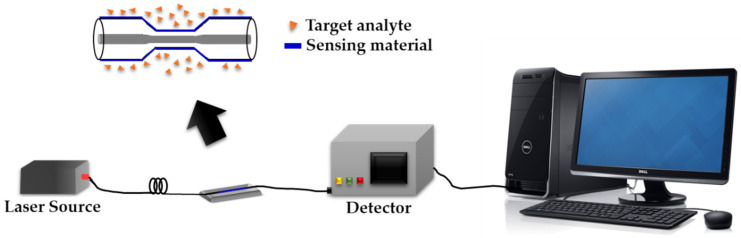
Experimental setup for optical fiber sensor–absorbance mode.

Apart from increasing light interaction with the target analyte, studies also show that by integrating the sensing material with a microfiber offers more binding sites for analyte attachment, and due to this reason, the sensor sensitivity and selectivity are greatly improved. Figure 13 and Figure 14 show the microfiber experimental setup in reflectance and absorbance mode, respectively. 

#### 4.4.2. Surface Plasmon Resonance-Based Optical Fiber Sensors Using Polysaccharide-Based Materials

**Figure 15:**
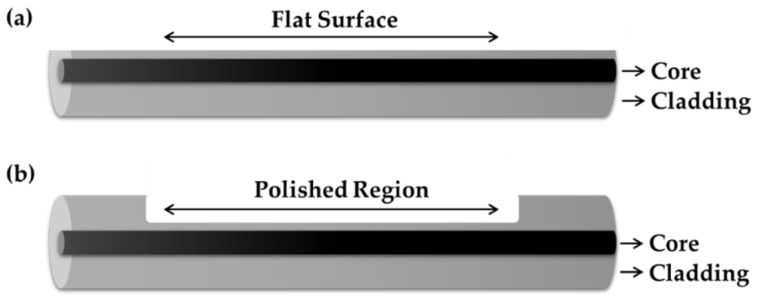
Structure of (**a**) D-shaped and (**b**) side-polished optical fibers.

There are two ways to perform a surface plasmon resonance technique using optical fibers. One can use a d-shaped optical fiber, where the cladding is removed entirely from one side of the fiber (Figure 15a). Another way is by polishing a small region of the fiber surface on one side of the fiber, however, for this type of fiber, the cladding is not removed entirely (Figure 15b). To initiate the plasmonic effect, a metallic thin film, such as Au or Ag, is applied on the flat surface or on the polished region of the optical fiber prior to sensing material deposition. 

Verma and Gupta (2015) [78] developed an SPR-based optical fiber for the detection of Cd^2+^, Pb^2+^ and Hg^2+^ using a pyrrole/CS composite as the active sensing layer. The optical fiber surface was coated with Ag and indium tin oxide (ITO) prior to pyrrole/CS composite deposition. By adding an additional layer of high index dielectric metal, such as ITO, followed by the immobilization of the polymer coating, enabled the enhancement of the sensor sensitivity. They reported that the pyrrole/CS/Ag/ITO probe was highly sensitive and selective towards Cd^2+^ compared to the other metal ions under testing, with sensitivity and detection limits of 2.589 nm nM^−1^ and 0.256 ppb, respectively. 

**Figure 16:**
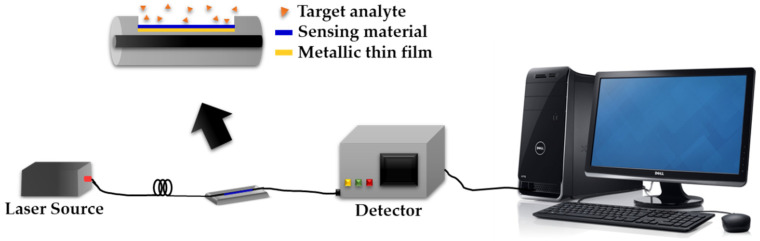
Experimental setup for optical fiber sensor based on SPR technique.

Recently, Sadani and co-workers [79] developed a localized SPR based on a U-bend optical fiber sensor technique for the highly selective detection of Hg^2+^ in various samples. In their work, a combination of a sensing material of CS-capped AuNPs coated on bovine serum albumin (BSA) was used. The combination of these sensing materials offers more adsorption sites for Hg^2+^ binding, such as on the nitrogen, oxygen and sulfur atoms of CS and BSA. It is also reported that the hydrophobic interactions occurred between Hg^2+^ and the alpha helix of BSA [79]. Consequently, a low detection limit of about 0.1 ppb was obtained, which is 20 times lower than the Environmental Protection Agency (EPA) standard guidelines [79]. Figure 16 shows the experimental setup for an optical fiber sensor based on the SPR technique.

#### 4.4.3. Interferometry-Based Optical Fiber Sensors Using Polysaccharide-Based Materials

Another optical fiber sensor technique is interferometry, where this technique measures the interference that occurs during the optical sensor analysis. High sensitivity and stability, large dynamic range and compact structure are some of the advantages of this technique [45]. There are four main types of interferometers, which are classified based on their configuration setup, such as the Mach–Zehnder interferometer (MZI) [42,80], Michelson interferometer (MI) [45], Sagnac interferometer (SI) [45] and Fabry–Perot interferometer (FPI) [43,81]. Each different interferometric technique possesses its own advantages and disadvantages, hence producing different sensing performances.

Ragunandhan and co-workers [45] developed an interferometric optical fiber sensor technique based on MZI using CS/polyacrylic acid (PAA) as a sensing material for Ni^2+^ ion detection. They used a no-core fiber and coated the CS/PAA on the surface of a no-core region of the optical fiber. The sensing principle is based on the interference of the core and cladding modes to detect changes in the refractive index induced by Ni^2+^ adsorption on the functionalized sensor. The reported sensitivity and detection limits are 0.0554 nm µM^−1^ and 0.167 µM, respectively. In another report, Ni and co-workers [42] developed a humidity interferometric optical fiber sensor based on an MZI configuration using CS as the sensing material. Using a single mode fiber (SMF), the sensor probe was fabricated by the fusion splicing of a segment between the two SMFs with waist-enlarged fusion bitapers and followed by CS coating. The reported sensitivity was approximately 119.6 pm RH^−1^. Another work, which also used an MZI configuration, was reported by Lin and co-workers [80]. They utilized a multilayer film of CS/multi-walled carbon nanotubes (MWCNT)/PAA for the detection of Ni^2+^. A high sensitivity of 56.5 dM/mM was obtained for Ni^2+^ detection at concentrations ranging from 0.3 to 0.7 mM. 

Recently, an interferometric technique based on the Fabry–Perot technique using CS for metal ion sensing was reported by Yulianti and co-workers [43]. According to their report, CS has a higher affinity towards Hg^2+^ compared to that of Pb^2+^ and Ni^2+^. This is due to the higher atomic radius possessed by Hg^2+^, which is 171 pm, compared to Pb^2+^ and Ni^2+^, with 154 and 149 pm, respectively. The sensitivity reported was −0.215 dBm ppm^−1^, −0.177 dBm ppm^−1^ and 0.145 dBm ppm^−1^, respectively. Another work that used a combination of CS with the Fabry–Perot technique for the detection of humidity was reported by Chen and co-workers [81]. The sensor showed a fast response time of 380 ms with a sensitivity of 0.13 nm/%RH.

In a nutshell, apart from integrating sensing materials with optical fibers for the enhancement of sensor performance, researchers also manipulated the optical fiber structure in numerous ways to improve the sensing performance and applied it in various applications. Table 5 shows the summary of optical fiber sensor techniques based on polysaccharides as sensing materials.

Although there are many advantages of using polysaccharides as sensing materials in optical sensor applications, there are apparently no commercial sensors using chitosan or carrageenan as sensing layers to date. Most of the available sensors using polysaccharides are still in the pre-commercialization stage. The limitations that lead to this issue are mostly related to the properties of polysaccharides, such as adsorption capacity limitations, stability and selectivity issues for long-term usage [82]. On the other hand, since polysaccharides possess slippery properties, the attachment of polysaccharides to thin films on the surface of sensing substrates, such as glass substrate or optical fibers, is another issue that needs to be highlighted. Although such limitations can be improved through the modification of polysaccharides, from the author’s point of view, the study of long-term stability and sensing material attachment on sensor surfaces need to be further studied. 

## 5. Summary

In summary, polysaccharides are widely applied in optical sensor applications due to their bio-based nature and remarkable features. These two materials possess many functional groups that are able to interact with target analytes. For instance, chitosan has O-H, N-H and C-O functional groups, while carrageenan possesses O=S=O, O-H and C-O functional groups. Interestingly, these materials are robust, where they can be designed according to the needs of application. The incorporation of inorganic materials, metal nanoparticles, graphene oxides and quantum dots with polysaccharides produces polysaccharide composites. Meanwhile, the addition of organic polymers into polysaccharide structures produces polysaccharide blends. Polysaccharide blends are prepared in two ways: (i) via solution blending and (ii) melt blending methods. Both polysaccharide composites and polysaccharide blends are prepared through physical modification. As for chemical modification, the structure of polysaccharides is altered by chemical reactions, either by adding or substituting a specific functional group, such as carboxymethyl, succinyl or phosphonyl in its structure. In general, the modification of polysaccharides either by physical or chemical means tremendously improved the physicochemical properties of polysaccharides, which is essential for optical sensor applications. Another intriguing feature of polysaccharides is their ability to interact with analytes in many ways, such as through chelation, electrostatic interaction and hydrogen bonding. These interactions are dependent on the properties and availabilities of functional groups or chemical substances that are present in polysaccharides, as well as analytes. The integration of polysaccharides with optical sensing techniques is crucial to further improve optical sensor performance. Both of them can be integrated through several techniques: (i) dip-coating, (ii) spin-coating and (iii) layer-by-layer assembly (LBL). To achieve a well-distributed coating material, the dip-coating technique is suitable to be used for both flat surface glass substrates and optical fibers, while the spin-coating technique is normally used for flat surface glass substrates and the LBL technique is suitable for optical fibers. Polysaccharides have been applied in various optical sensing techniques, such as SPR, LSPR and optical fiber sensor techniques. The application of polysaccharides as sensing materials is significant in environmental applications, particularly for heavy metal and dye sensing. Although the use of polysaccharides is superior in terms of their bio-based nature and remarkable features, there are also some limitations to using this material as a sensing element in sensor applications, such as long-term stability and sensing material attachment on the sensor surface. From the authors’ point of view, this issue is important for commercialization, as it affects the long-term usage and stability of the sensors produced. 

## Figures and Tables

**Table 1:** List of natural polysaccharides and their origins [10].

Origin	Polysaccharides
Algae	Carrageenans, alginates, agar, galactans
Fungi	Chitin, chitosan, elsinan, pullulan, yeast glucans
Bacteria	Cellulose, dextran, xanthan, gellan, polygalactosamine
Plants	Starch, cellulose, hemicellulose, glucomannan, gums
Animals	Chitin, chitosan, cellulose, hyaluronic acid, glycosaminoglycans

**Table 2:** Summary of colorimetric optical sensing performance using polysaccharides.

Sensing Material	Target Analyte	Color Changes	Sensitivity	LOD	Ref.
Carboxymethyl Chitosan/QDs	Zn^2+^ Lysozyme	- -	- -	4.5 μM 0.031 ng mL^−1^	[49] [50]
Carboxymethyl Chitosan/Quercetin	Al^3+^	-	0.187 Abs^−1^ mg mL^−1^	-	[51]
Chitosan/AuNP/GO	NO_2_^-^	Wine Red-Purple	-	0.1 μM	[52]
Chitosan/AuNP@S-g-C_3_N_4_	Hg^2+^	Wine Red-Colorless	-	0.275 nM	[53]
Carrageenan/Ag/AgCl	Hg^2+^	Dark Brown-White	0.00318	1 μM	[54]
Carrageenan/AgNP	Hg^2+^	Dark Brown-White	-	2.92 × 10^−4^ M	[55]
	Cu^2+^ S^2-^	Yellow-Colorless Yellow-Brown	-	1.7 μM 2 μM	[56] [56]

**Table 3:** Summary of surface plasmon resonance (SPR) techniques based on polysaccharides.

Sensing Material	Metallic Layer	Target Analyte	Sensitivity	Ref.
Chitosan	Au	Fe^3+^	0.015 Δθ ppm^−1^	[62]
Crosslinked chitosan	Au	Hg^2+^ Cu^2+^	0.007 ppm^−1^ 0.006 ppm^−1^	[63]
Chitosan/p-tert-butylcalix [4] arene-tetrakis	Au	Pb^2+^	0.045° ppm^−1^	[64]
Chitosan/tetrabutyl thiuram disulphide	Au	Zn^2+^	0.032° ppm^−1^	[65]
Chitosan	Au	Pb^2+^	0.776 ppm^−1^	[66]
Chitosan/graphene oxide	Au	Pb^2+^	1.112 ppm^−1^	[66]
Chitosan/graphene oxide	Ag/Au	Pb^2+^	-	[67]
Chitosan/graphene oxide	Ag/Au/Ag	Pb^2+^	1.332 ppm^−1^	[7]
Chitosan/graphene oxide	Ag	Pb^2+^	1.380° ppm^−1^	[3]
Chitosan/OH-GQDs	Au	Fe^3+^	0.1139° ppm^−1^	[68]
Chitosan/COOH-GQDs	Au	Hg^2+^	0.0006° ppm^−1^	[69]

**Table 4:** Summary of LSPR techniques based on polysaccharides.

Sensing Material	Metal Nanoparticle	Target Analyte	Sensitivity	LOD	Ref.
Chitosan	Au	Fe^3+^ Cu^2+^	- -	0.5 µM 0.5 mM	[74]
Chitosan	Au	Cr^6+^	0.022 nm ppm^−1^	10 ppm	[75]
Chitosan	Au	Pb^2+^	0.8165 nm ppm^−1^	-	[1]
Carrageenan	Au	Pb^2+^	1.3535 nm ppm^−1^	-	[1]

**Table 5:** Summary of optical fiber sensor techniques based on polysaccharides.

Sensing Material	Optical Fiber Sensor Technique	Target Analyte	Sensitivity	LOD	Ref.
Chitosan	Microfiber	Pb^2+^	40.550 abs ppm^−1^	-	[11]
Pyrrole/Chitosan/ITO/Ag	SPR	Cd^2+^ Pb^2+^ Hg^2+^	2.589 nm nM^−1^ 2.101 nm nM^−1^ 1.135 nm nM^−1^	0.256 ppb 0.440 ppb 0.796 ppb	[78]
Chitosan/BSA/Au	SPR U-bent	Hg^2+^			[79]
Chitosan/PAA	MZI	Ni^2+^	0.0554 nm µM^−1^	0.167 µM	[45]
Chitosan	MZI	H_2_O	119.6 pm RH^−1^	-	[42]
Chitosan/MWCNT/PAA	MZI	Ni^2+^	56.5 dB/mM	-	[80]
Chitosan	Fabry Perot	Hg^2+^ Pb^2+^ Ni^2+^	−0.215 dBm ppm^−1^ −0.177 dBm ppm^−1^ 0.145 dBm ppm^−1^	- - -	[43]
Chitosan	Fabry Perot	H_2_O	0.130 nm/RH%	-	[81]
